# Major histocompatibility complex class II DAB alleles associated with intestinal parasite load in the vulnerable Chinese egret (*Egretta eulophotes*)

**DOI:** 10.1002/ece3.2226

**Published:** 2016-06-07

**Authors:** Wei Lei, Xiaoping Zhou, Wenzhen Fang, Qingxian Lin, Xiaolin Chen

**Affiliations:** ^1^ Key Laboratory of Ministry of Education for Coast and Wetland EcosystemsCollege of the Environment and EcologyXiamen UniversityXiamen361102China

**Keywords:** Ecological genetics, major histocompatibility complex diversity, natural selection, parasite load, wild bird

## Abstract

The maintenance of major histocompatibility complex (MHC) polymorphism has been hypothesized to result from many mechanisms such as *rare‐allele advantage*,* heterozygote advantage*, and *allele counting*. In the study reported herein, 224 vulnerable Chinese egrets (*Egretta eulophotes*) were used to examine these hypotheses as empirical results derived from bird studies are rare. Parasite survey showed that 147 (65.63%) individuals were infected with 1–3 helminths, and 82.31% of these infected individuals carried *Ascaridia* sp. Using asymmetric polymerase chain reaction technique, 10 DAB1, twelve DAB2, and three DAB3 exon 2 alleles were identified at each single locus. A significant association of the rare allele *Egeu*‐DAB2*05 (allele frequency: 0.022) with helminth resistance was found for all helminths, as well as for the most abundant morphotype *Ascaridia* sp. in the separate analyses. *Egeu*‐DAB2*05 occurred frequently in uninfected individuals, and individuals carrying *Egeu*‐DAB2*05 had significantly lower helminth morphotypes per individual (HMI) (the number of HMI) and the fecal egg count values. Further, the parasite infection measurements were consistently lower in individuals with an intermediate number of different alleles in the duplicated DAB loci. Significantly, heterozygosity within each DAB locus was not correlated with any parasite infection measurements. These results indicate that the diversity in MHC *Egeu*‐DAB gene is associated with intestinal parasite load and maintained by pathogen‐driven selection that probably operate through both the *rare‐allele advantage* and the *allele counting strategy*, and suggest that *Egeu*‐DAB2*05 might be a valuable indicator of better resistance to helminth diseases in the vulnerable Chinese egret.

## Introduction

The major histocompatibility complex (MHC), a multigene family, plays an important role in susceptibility and/or resistance to many vertebrate diseases, principally by recognizing foreign peptides and presenting them to T cells of the immune system, thereby initiating the adaptive immune response (Klein 1986; Klein et al. 1993; Frank 2002; Sommer 2005). MHC genes are also valuable genes for the studies of evolutionary and conservation biology because of their diverse functions and characteristics relevant to evolutionary and adaptive processes (Hess and Edwards 2002; Sommer 2005; Piertney and Oliver 2006; Alcaide et al. 2008; Radwan et al. 2010b; Eizaguirre et al. 2012).

Traditionally, this multigene family is classified into two major classes: class I and class II. MHC class II genes can be further subdivided into A and B genes. DR genes (e.g., DAB), members of the B gene family, possess a high level of polymorphism and are highly variable among different species. Class II proteins deal with extracellular pathogens (e.g., bacteria or helminth), while MHC class I proteins are involved in response to intracellular pathogens (e.g., virus). Polymorphism of MHC proteins is related to the diversity of T‐lymphocyte receptors that in turn determine the pathogen resistance to an organism, because the MHC encodes cell surface glycoproteins that bind antigens derived from pathogens and present them to T lymphocytes to initiate the immune response (Klein 1986; Sommer 2005). MHC polymorphism is postulated to be generated by intra‐ and interlocus recombination or gene conversion, and the accumulation of *de novo* mutations (Ohta 1991; Nei and Rooney 2005; Li et al. 2011). Classical MHC genes have the highest levels of polymorphism known in vertebrates, especially in the functionally important peptide‐binding region (PBR) that is characterized by high levels of variation in both the number of alleles and the extent of sequence divergence between alleles (Hughes and Hughes 1995; Hughes and Yeager 1998; Bernatchez and Landry 2003; Harf and Sommer 2005; Schad et al. 2005). More nonsynonymous than synonymous substitutions in the PBR is strong evidence for positive selection driving MHC polymorphism (Klein 1986; Hughes and Nei 1988; Jeffery and Bangham 2000). The maintenance of allelic polymorphism in genes of the MHC is a central issue in evolutionary ecology and conservation genetics. Several hypotheses of pathogen‐driven selection underlying the maintenance of MHC polymorphism have been documented (Hedrick 2002; Penn 2002; Bernatchez and Landry 2003; Harf and Sommer 2005; Sommer 2005; Piertney and Oliver 2006; Alcaide et al. 2014).

The *rare‐allele advantage hypothesis* (also designated the *negative frequency‐dependent selection hypothesis*) postulates that rare alleles have a selective advantage over common alleles (e.g., Clarke and Kirby 1966; Bodmer 1972; Takahata and Nei 1990). According to this hypothesis, if some rare alleles are an advantage to the host, these resistant alleles will spread through the population. When the rare alleles become common, pathogens may evolve to escape the recognition by these alleles. This negative frequency‐dependent co‐evolutionary process between hosts and pathogens maintains MHC polymorphism within a population (Jeffery and Bangham 2000; Westerdahl et al. 2012; Zhang et al. 2015). In past, the rare‐allele advantage hypothesis has been well supported by evidence for the associations between particular alleles and susceptibility/resistance to infection, in a wide range of vertebrate taxa (e.g., Paterson et al. 1998; Godot et al. 2000; Langefors et al. 2001; Schad et al. 2005, 2012; Kloch et al. 2010; Zhang and He 2013; Kamath et al. 2014; Sin et al. 2014; Zhang et al. 2015) including some bird species, including *Passer domesticus* (Loiseau et al. 2008), *Geothlypis trichas* (Dunn et al. 2013), *Cyanistes caeruleus* (Westerdahl et al. 2013) and *Zonotrichia capensis* (Jones et al. 2015).

The *heterozygote advantage hypothesis* predicts that heterozygous individuals will show lower levels of infection, as they express more MHC alleles and thus can resist a broader spectrum of pathogens than homozygotes (Doherty and Zinkernagel 1975). The most convincing evidence for this hypothesis is that heterozygotes of MHC‐congenic *Mus musculus* are found to be favoured, because of a significant superiority of heterozygotes to homozygotes against multiple pathogens (McClelland et al. 2003). However, up to the present, there are only a few studies supporting this hypothesis that mainly focus on mammals and fish (e.g., *Rhabdomys pumilio*, Froeschke and Sommer 2005; *Oncorhynchus tshawytscha*, Evans and Neff 2009; *Arvicola terrestris*, Oliver et al. 2009; *Canis lupus*, Niskanen et al. 2014; *Phocarctos hookeri*, Osborne et al. 2015).

The *allele counting hypothesis* suggests that an intermediate number of alleles rather than a maximal number is optimal for an individual (Reusch et al. 2001). Although high intraindividual allele diversity should be directed toward recognizing a broader array of pathogens, individuals with too many different MHC alleles will cause a greater risk of autoimmune disease or net loss of the mature T‐cell repertoire during the thymic selection process (Nowak et al. 1992; Harf and Sommer 2005; Milinski 2006; Woelfing et al. 2009). This allele counting “strategy” for optimizing the immunocompetence has been confirmed in studies on *Gasterosteus aculeatus* (Reusch et al. 2001; Wegner et al. 2003a,b) and *Myodes glareolus* (Kloch et al. 2010), but not in some bird studies (e.g., Bonneaud et al. 2004; Radwan et al. 2012; Dunn et al. 2013).

Most of empirical evidences in support of these three hypotheses have been derived from the studies conducted in mammals, fish, and birds, or carried out under experimental laboratory conditions (e.g., Reusch et al. 2001; McClelland et al. 2003; Worley et al. 2010; Eizaguirre et al. 2012; Rivero‐de Aguilar et al. 2016). There is, however, a dearth of endangered bird studies on testing the hypotheses by using intestinal parasite load as an indicator under natural conditions in particular (Bernatchez and Landry 2003; Harf and Sommer 2005; Niskanen et al. 2014; Sin et al. 2014; Osborne et al. 2015). Generally, the levels of MHC variation in endangered species exhibit lower than those in common species (Marsden et al. 2009; Radwan et al. 2010a), and low MHC polymorphism may be related to higher susceptibility to infectious disease (O'Brien and Evermann 1998; Sommer 2005; Radwan et al. 2010a,b).

**Figure 1:**
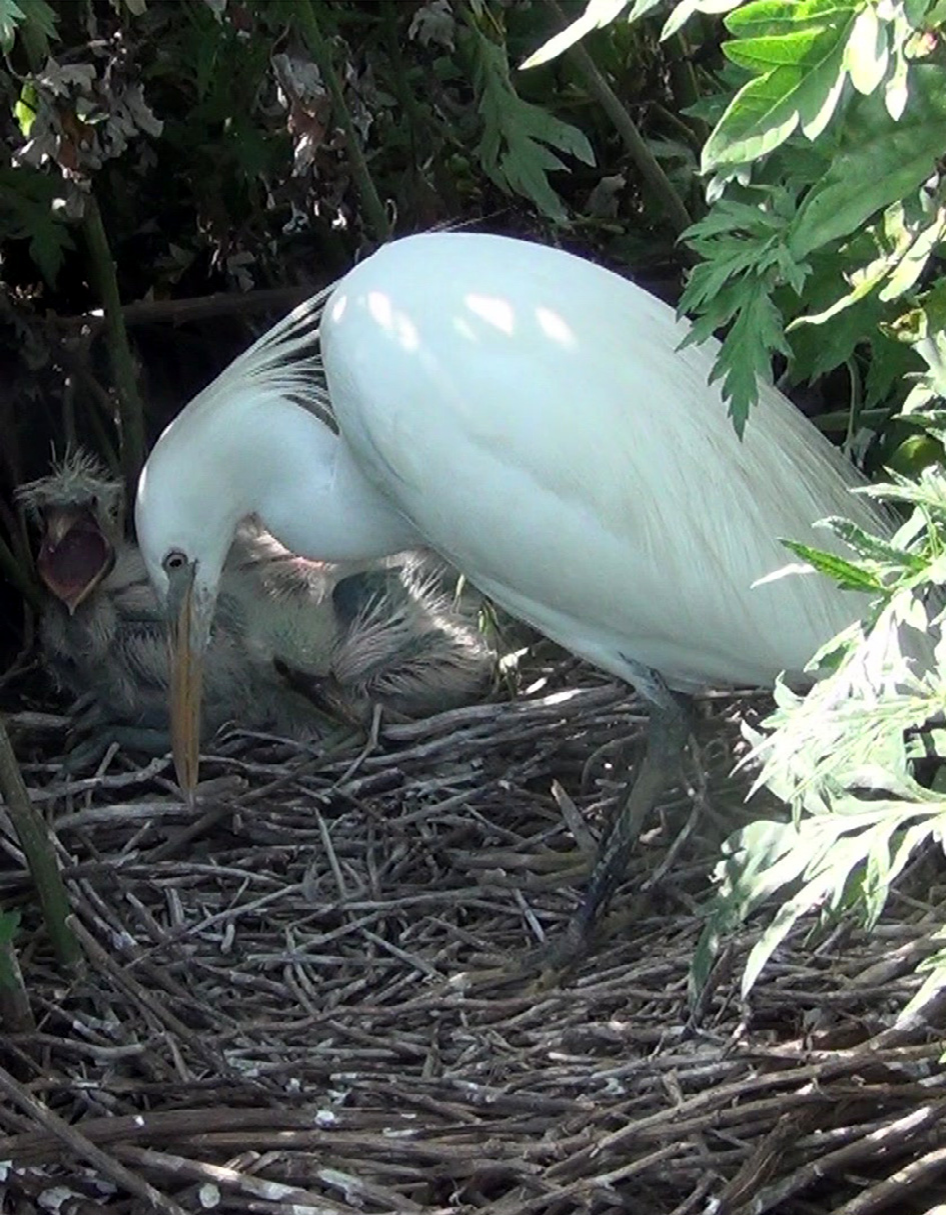
The Chinese egret (*Egretta eulophotes*) in breeding.

The Chinese egret (Ciconiiformes, Ardeidae, *Egretta eulophotes*) (Fig. 1) is a species of migratory colonial waterbird whose populations have been declining dramatically since the nineteenth century (Kushlan and Hancock 2005). Currently, this bird is listed as a vulnerable species with an estimated global population of 2600–3400 individuals. The migratory colonial life pattern wintering in the south of Asia while breeding on offshore islands in Russia, North Korea, South Korea, and China may facilitate the pathogen transmission and advance MHC polymorphism in this species (Shiina et al. 2004; BirdLife International 2015; IUCN 2015). Recently, some evidences indicate that parasites have adverse effects on the life and survival of many migratory waterbirds, especially endangered ones (Poulin 1999; Huang et al. 2014). The migratory waterbirds might suffer more immunological suppression and infection risk from parasites than other birds during migration and aggregation (Huang et al. 2014). In our previous MHC studies on this vulnerable species, we isolated and characterized three classical single‐copy loci of MHC class II DAB gene (named *Egeu‐*DAB1, ‐DAB2, and ‐DAB3) and established an efficient locus‐specific MHC genotyping technique (Li et al. 2011; Wang et al. 2013; Lei et al. 2015). Our genetic diversity study on this egret showed that there was a relatively high level of mitochondrial DNA genetic diversity in three populations in China, and found that these populations had low but significant genetic differentiation with little geographical structure (Zhou et al. 2010). Because little is known concerning the intestinal parasite, and the association between parasite load and *Egeu‐*DAB gene in the vulnerable Chinese egret, we initiated the present study to address the following three specific aims: (1) to investigate the levels of parasite load in this species under natural conditions; (2) to explore the importance of the constitutions of individual DAB exon 2 for resistance to parasites; and (3) to determine what selective mechanisms might be acting on *Egeu‐*DAB in the presence of parasites. Successful completion of these aims will provide the essential fundamentals for further understanding the mechanism of MHC adaptive evolution in the vulnerable Chinese egret and other birds.

## Materials and Methods

### Sample collection

Sample collection of the Chinese egret was conducted during the morning on Xingrentuo Islet (39°31′N, 123°03′E) in Liaoning Province of China during 2012 and 2013. Visits to the breeding colony were restricted to a maximum of 2 h per day, and the individual birds were immediately returned to their nests after sampling. All procedures involving the collection of animal tissue in the wild were approved by the Administration Center for Wildlife Conservation in Fujian Province (FJWCA‐1208) and were carried out in accordance with their ethical standards. Intestinal parasite load and *Egeu‐*DAB variability were examined in a total of 224 Chinese egret nestlings. For parasite survey, spontaneously excreted feces were individually collected from these hand‐captured nidicolous birds (10–15 days old) from their nests. Feather samples were also collected, and then, any bleeding was stanched with cotton. All samples were immediately preserved in 95% ethanol.

### Parasitic screening

The McMaster flotation egg counting technique (Sloss et al. 1994), modified by Meyer‐Lucht and Sommer (2005), was applied for the identification and quantification of parasite species. This technique has been established as an efficient method for the quantification of helminth eggs in many recent studies (e.g., Froeschke and Sommer 2005; Harf and Sommer 2005; Meyer‐Lucht and Sommer 2005; Schad et al. 2005; Schwensow et al. 2007; Kamath et al. 2014; Valilou et al. 2015). Feces were screened for helminth eggs by counting two chambers of McMaster for each sample, and using a flotation dilution of potassium iodide with a specific density of 1.5 g/mL (Meyer‐Lucht and Sommer 2005). Helminth eggs were assigned to morphotypes based on the size and morphological characteristics. The number of different helminth morphotypes per individual (HMI) and the individual fecal egg count (FEC, eggs per gram of feces) were used as measurements for parasite load. These two noninvasive measurements are expressive indicators that reflect worm burden and the immune state of the host (Stear et al. 1997; Paterson and Viney 2002).

### Molecular biological techniques

Genomic DNA (gDNA) was isolated from the feather samples using the Universal Genomic DNA Extraction Kit Ver. 3.0 (TaKaRa, Dalian, China) following the manufacturer's protocols. Genetic polymorphism within exon 2 sequences of the three single DAB loci was separately examined by semi‐nested asymmetric polymerase chain reaction (PCR) combined with single‐strand conformation polymorphism (SSCP). To specially amplify the three single DAB loci, a first‐round PCR was carried out in a final volume of 20 μL, which contained 1 μL (approximately 100 ng) gDNA, 0.7 U of Taq polymerase (TaKaRa), 1.5 mmol/L MgCl_2_, 200 μmol/L of each dNTP, and 0.4 μmol/L of each primer. The three forward locus‐specific primers DAB01F, DAB02F (Wang et al. 2013), and DAB03F1 (Lei et al. 2015) were combined with the reverse primer DAB2exR (Wang et al. 2013) to amplify the three loci, respectively. Thermocycling conditions were as follows: 94°C for 3 min, 25 amplification cycles at 94°C for 30 sec, 60°C for 30 sec, and 72°C for 60 sec, final extension at 72°C for 10 min. To obtain the suitable length fragments for SSCP genotyping, second‐round PCR was conducted on each respective sample, using the primer set DAB2exR and DAB2exF (Wang et al. 2013), which could amplify the entire exon 2 (270 bp) in each locus. PCR products diluted 40‐fold from the first round were used as the template for second‐round PCR. The reaction conditions for the second‐round PCR were identical with those described for the first round. To produce the single‐stranded amplicons, a third‐round PCR, asymmetric PCR, was performed. The reaction conditions for the third‐round PCR were same as those in the second round except for using only one primer DAB2exR and using second‐round PCR products as template. The single‐stranded amplicons were loaded on 10% nondenaturing polyacrylamide gels (PAGEs) and, after electrophoresis (240 V at 5°C for 16 h), visualized by the sensitive silver staining procedure. Finally, SSCP bands were excised from the gels, re‐amplified, and sequenced following the protocols of Wang et al. (2013). To avoid the inclusion of PCR artifacts, every allele was directly sequenced in both directions from at least two different individuals or two independent PCRs from one individual. Throughout this study, the word “allele” is used to describe a 270‐bp exon 2 sequence derived from SSCP genotyping. In addition, the sex of examined individuals was determined following the protocols of Wang et al. (2011).

### Data analyses

Exon 2 sequences obtained from the 224 individuals were aligned and edited using BioEdit v7.0.5.3 (Hall 1999). Estimates of allele frequency, the effective number of alleles, observed heterozygosity and expected heterozygosity, and tests of deviation from Hardy–Weinberg equilibrium were assessed using GENEPOP 4.0 (Rousset 2008). Calculations of nucleotide diversity and gene diversity were made in Arlequin 3.5 (Excoffier and Lischer 2010) and FSTAT 1.2 (Goudet 1995), respectively. Further, positive selection, evidenced by a significantly higher number of nonsynonymous substitutions per nonsynonymous codon site (*d*_N_) relative to synonymous substitutions per synonymous codon site (*d*_S_), was determined. The *Z*‐test implemented in MEGA 6 (Tamura et al. 2013) was carried out to compare *d*_N_ with *d*_S_ at all sites, PBR sites as defined by Brown et al. (1993), and non‐PBR sites for each DAB locus. Standard errors (SE) were based on 1000 bootstrap replications, including average rates of nonsynonymous and synonymous substitutions per site using the Nei–Gojobori method with Jukes–Cantor correction for multiple substitutions (Nei and Gojobori 1986).

For the parasite analyses, we calculated the parasitic diversity, described by richness (the total number of parasite species), diversity (using the Shannon–Wiener index), and evenness (using the Pielou index) (Huang et al. 2014). FEC values were transformed to log_10_ (egg count + 1) to produce approximately normal distributed data. To assess the relative risk of being infected, the odds ratio test was carried out using a 2 × 2 cross classification table. This test is a common test in epidemiological studies evaluating the exposition of individuals carrying a risk factor (Sachs 1992). The allelic divergence in heterozygous individuals was calculated as the number of differing amino acids between the two alleles (Meyer‐Lucht and Sommer 2005). All statistical analyses were performed with SPSS software, version 17.0 (SPSS Inc., Chicago, IL). Data were presented as means ± SE, calculations were two‐tailed, and significance was accepted at the 0.05 probability level. The sequential Bonferroni procedure was applied where appropriate to keep the type I error levels at *α *≤ 0.05 (Sachs 1992).

## Results

### Parasite load

In the 224 Chinese egret individuals examined, 10 distinct helminth egg morphotypes were identified. These distinct morphotypes included four nematode species, four trematode species, one cestode species, and one coccidium species (Table 1). The Shannon–Wiener index and Pielou index were 1.14 and 0.50, respectively. The HMI varied between no infection (34.37%) and one (68.71%), two (26.53%), or three (4.76%) different HMI. The most abundant helminth morphotype *Ascaridia* sp. appeared in 54.02% of all examined individuals (*n* = 224), while the remaining nine morphotypes were found in 0.45% to 22.77% in all individuals. The *Ascaridia* sp. occurred in 82.31% of the infected individuals (*n *=* *147) and accounted for 82.90% of the total FEC rate. Further analyses were calculated for all helminths and the most abundant morphotype *Ascaridia* sp. separately.

**Table 1:** Percentage of infected individuals and the number of helminth morphotypes in the 224 Chinese egrets examined

Helminth	No. of infected individuals	Infected (%)	No. of morphotypes
Overall	147	65.63	10
Nematode	125	55.80	4
Trematode	59	26.34	4
Cestode	9	4.02	1
Coccidium	7	3.13	1

To test the effect of year (2012: 130 individuals; 2013: 94 individuals) or sex (males: *n *=* *107; females: *n *=* *117) on the parasite load of the Chinese egret individuals, a generalized linear model with both year and sex was constructed. The results showed that both year and sex did not influence the infection status (all helminths or only *Ascaridia* sp.: all *P *>* *0.05), the HMI values (both *P *>* *0.05), or the FEC values (all helminths or only *Ascaridia* sp.: all *P *>* *0.05). Therefore, in Table 1, data of different years and sexes were combined for analyses.

### Major histocompatibility complex variability

**Figure 2:**
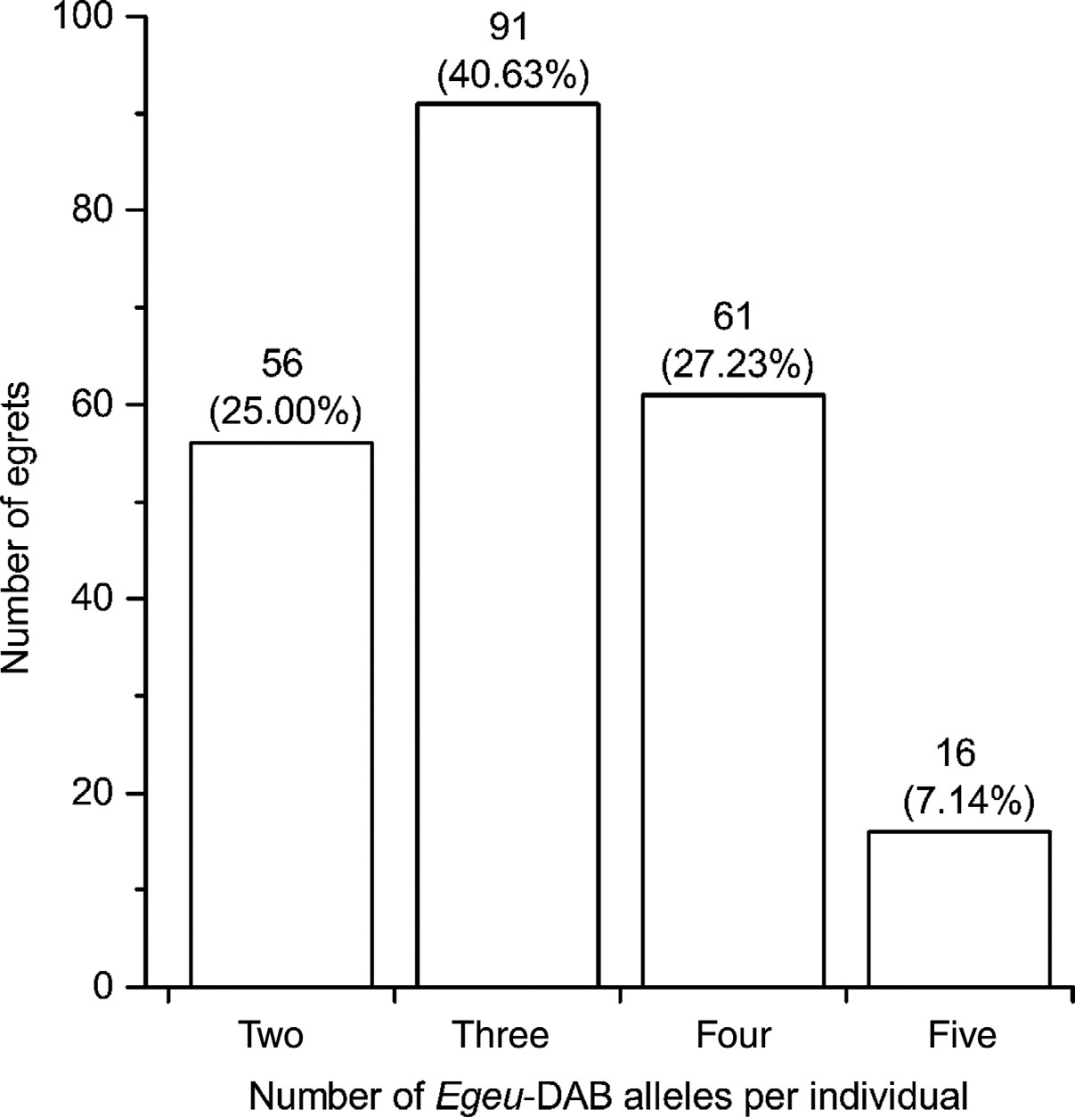
Frequency distribution of the number of different alleles in the duplicated DAB loci per individual. Number of egrets and their proportion (in parentheses) in total sample size (*n* = 224) are given above the bars. The mean number of *Egeu*‐DAB alleles is 3.17 ± 0.06.

Detailed MHC DAB exon 2 variability statistics are summarized in Tables 2 and 3. Of the 270 nucleotide positions in exon 2 of *Egeu*‐DAB1, 52 (19.26%) positions were variable, and 10 distinct alleles were identified. For *Egeu*‐DAB2, 49 of the 270 (18.15%) nucleotide positions were variable, and 12 distinct alleles were identified. For *Egeu*‐DAB3, 29 of the 270 (10.74%) nucleotide sites were variable, and three distinct alleles were identified (Tables 2 and S1). According to the nomenclature proposed by Klein et al. (1990), sequences of these confirmed alleles were denoted by the species' gene prefix (*Egeu*‐DAB) with a suffix comprising a locus number (1–3) and two sequential allele numbers (01–12) (available at GenBank, accession numbers: KP729234–KP729243, KP729246–KP729257, and KP729260–KP729262). Estimation of heterozygosity showed that all three loci exhibited significantly (*P *<* *0.05) lower levels of observed heterozygosity than expected (Table 2). The deviation from Hardy–Weinberg equilibrium within each locus was also statistically significant (*P *<* *0.001). In both *Egeu*‐DAB1 and ‐DAB2 loci, there were some rare alleles that occurred with a frequency of <0.05, that is, *Egeu*‐DAB1*04, *Egeu*‐DAB1*07–10, *Egeu*‐DAB2*03, and *Egeu*‐DAB2*05–12 (Table 3). For all three of these loci, always one to two alleles were identified per individual, suggesting that for each locus only one gene copy was sequenced with the primer sets. Fourteen pairs of identically shared alleles (e.g., *Egeu*‐DAB1*01 and *Egeu*‐DAB2*03) were found among the three single loci (Table 3 and Fig. S1), resulting in 3.17 (SE: 0.06, minimum: 2, maximum: 5) different alleles in the duplicated DAB loci per individual (Fig. 2).

**Table 2:** Summary of sequence variation of major histocompatibility complex (MHC) DAB exon 2 in the Chinese egret

Locus	Na	Ne	Ho	He	Sn	Saa	*π*	Gd
*Egeu*‐DAB1	10	3.937	0.308	0.748^a^	52	27	0.055	0.749
*Egeu*‐DAB2	12	2.436	0.442	0.591^a^	49	27	0.028	0.591
*Egeu*‐DAB3	3	1.426	0.152	0.299^a^	29	16	0.023	0.300

Observed (Na) and effective (Ne) number of alleles, observed (Ho) and expected (He) heterozygosities, the number of variable nucleotide (Sn) and amino acid (Saa) sites, nucleotide (*π*) and gene (Gd) diversities of the three MHC loci are indicated.

a Stands for significantly (*P *<* *0.05) lower levels of observed heterozygosity than expected.

**Table 3:** Allele frequencies and shared alleles of MHC DAB exon 2 in the Chinese egret

Allele\Locus	*Egeu*‐DAB1	*Egeu*‐DAB2	*Egeu*‐DAB3
01	0.230^C^	0.600^A^	0.828^I^
02	0.078^B^	0.176^B^	0.105^ J^
03	0.384^A^	**0.025** ^C^	0.067^D^
04	**0.007** ^F^	0.132	
05	0.085	**0.022** ^D^	
06	0.201^H^	**0.002** ^E^	
07	**0.002** ^ G^	**0.016** ^F^	
08	**0.002** ^E^	**0.002** ^ G^	
09	**0.004** ^I^	**0.011** ^H^	
10	**0.007** ^ J^	**0.004**	
11		**0.004** ^I^	
12		**0.004** ^ J^	

The frequencies of rare alleles (<0.05) are in *boldface*. The 14 pairs of identically shared alleles among the three loci are indicated by the letter (A–J, respectively) to the *right* of the frequencies.

Examining the amino acid sequences, 27 of 90 (30.00%) sites were variable for both *Egeu*‐DAB1 and ‐DAB2, whereas 16 of 90 (17.78%) sites were polymorphic for *Egeu*‐DAB3 (Table 2 and Fig. S1). All alleles of these three loci coded for unique amino acid sequences. Neither locus showed signs of frameshift mutations that would cause the alleles to become nonfunctional. For *Egeu*‐DAB1, ‐DAB2, and ‐DAB3 loci, the *d*_N_ values in the PBR were 5.32, 4.91, and 4.19 times higher than the *d*_N_ values in the non‐PBR, respectively. Significant signs of positive selection were found in all these three loci, with *d*_N_/*d*_S_ ratios of 2.451 (*Z *=* *2.697, *P *=* *0.004), 5.450 (*Z *=* *2.310, *P *=* *0.011), and 2.278 (*Z *=* *2.711, *P *=* *0.004), respectively. In contrast, all the ratios in the non‐PBRs of these loci did not significantly deviate from unity (all *Z*‐tests, *P *>* *0.05) (Table 4).

**Table 4:** Summary of nucleotide substitution rates of major histocompatibility complex (MHC) DAB exon 2 in the Chinese egret

Locus	Position	*d*_N_	*d*_S_	*d*_N_/*d*_S_	*Z*	*P*
*Egeu*‐DAB1	PBR	0.250 ± 0.062	0.102 ± 0.048	2.451	2.697	0.004
	Non‐PBR	0.047 ± 0.017	0.034 ± 0.019	1.382	0.577	0.282
	All	0.094 ± 0.020	0.052 ± 0.019	1.808	1.952	0.027
*Egeu*‐DAB2	PBR	0.221 ± 0.058	0.097 ± 0.050	2.278	2.310	0.011
	Non‐PBR	0.045 ± 0.015	0.032 ± 0.019	1.406	0.584	0.280
	All	0.086 ± 0.019	0.050 ± 0.019	1.720	1.772	0.039
*Egeu*‐DAB3	PBR	0.218 ± 0.078	0.040 ± 0.030	5.450	2.711	0.004
	Non‐PBR	0.052 ± 0.021	0.038 ± 0.023	1.368	0.460	0.323
	All	0.091 ± 0.024	0.039 ± 0.018	2.333	1.931	0.028

Rates of nonsynonymous (*d*_N_) and synonymous (*d*_S_) substitutions across all sites, sites of the putative peptide‐binding region (PBR) as defined by Brown et al. (1993), and non‐PBR of the three MHC loci are indicated. Standard errors are obtained through 1000 bootstrap replicates. Total size 270 bp (90 residues) for all sites, 72 bp (24 residues) for PBR sites, and 198 bp (66 residues) for non‐PBR sites. *P* is the probability that *d*_N_ and *d*_S_ are different by *Z*‐test.

### Test for the rare‐allele advantage hypothesis

**Figure 3:**
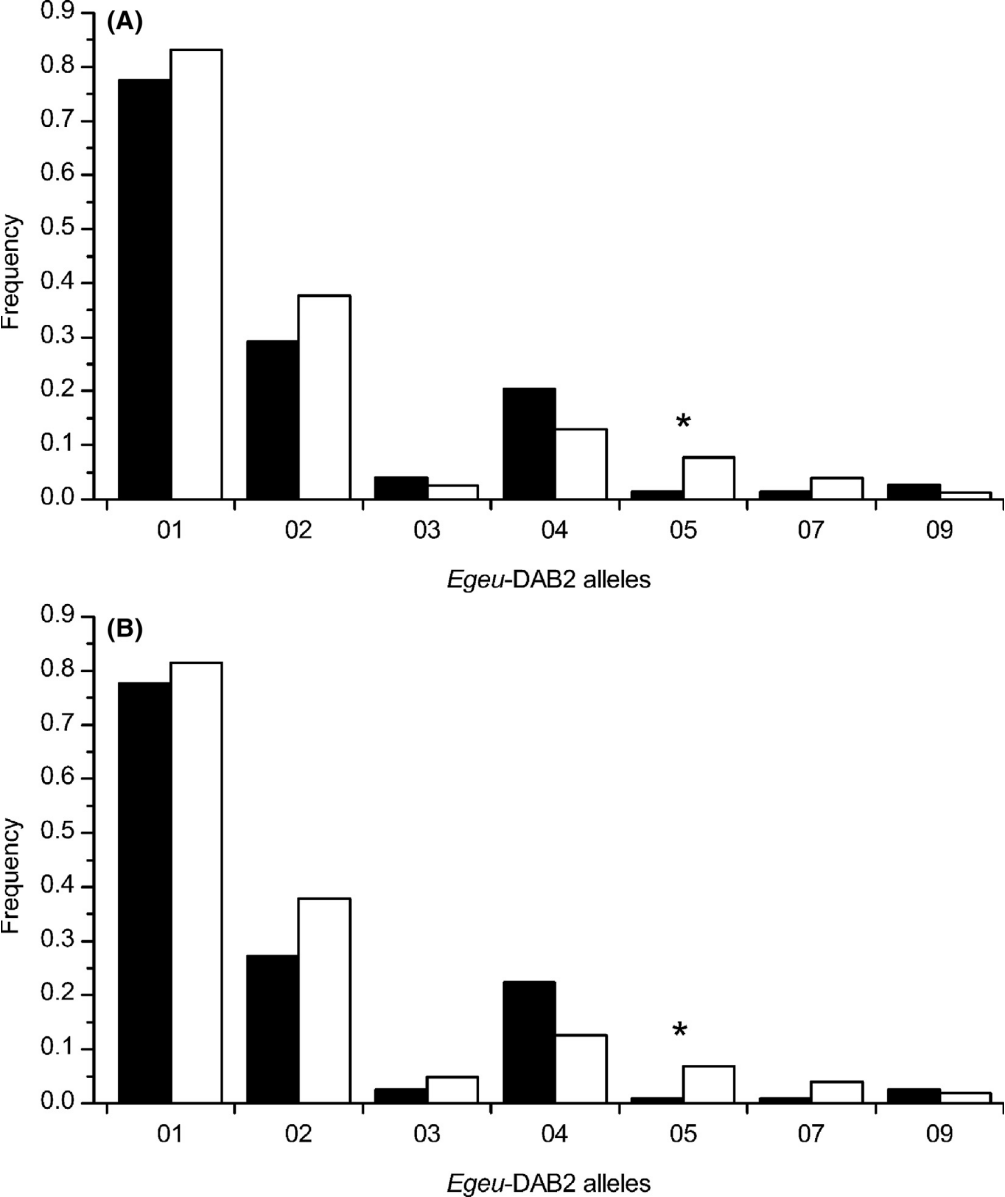
Frequencies of *Egeu*‐DAB2 alleles in infected (black bars) and uninfected (white bars) individuals. The frequencies are calculated with respect to (A) all helminths and (B) the most abundant morphotype *Ascaridia* sp. Alleles with low prevalence (<5 individuals) are not displayed. *P <* *0.05.

**Figure 4:**
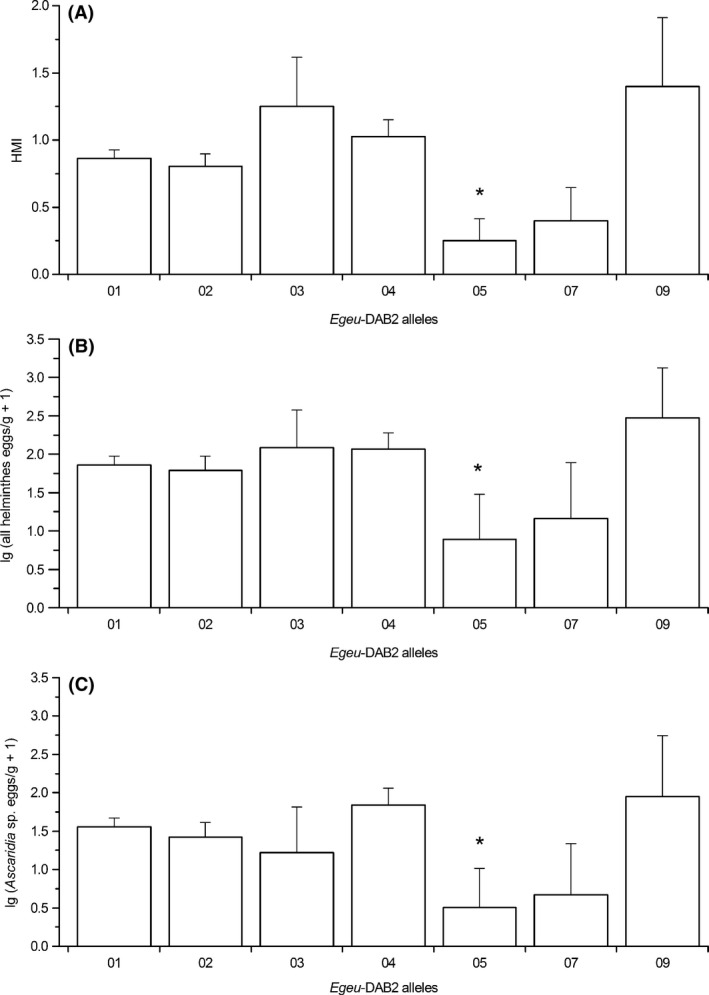
Associations of *Egeu*‐DAB2 alleles with the infection measurements. The infection measurements are calculated as (A) the number of helminth morphotypes per individual (HMI), the fecal egg counts (FEC, eggs/g) of (B) all helminths and (C) the most abundant morphotype *Ascaridia* sp. Means ± SE are given. Alleles with low prevalence (<5 individuals) are not displayed. *P <* *0.05.

To test for the *rare‐allele advantage hypothesis*, the three single loci were separately examined, and the effects of specific alleles on the individual parasite load were analyzed (Godot et al. 2000; Zhang et al. 2015). A positive relationship was observed between the allele *Egeu*‐DAB2*05 and parasite load, and no significant associations were found in all other alleles. *Egeu*‐DAB2*05 was significantly linked to uninfected individuals, irrespective of whether all helminths or only the most abundant morphotype *Ascaridia* sp. were considered (all helminths: *χ* ^2^ = 4.35, df = 1, *P* = 0.037, Bonferroni not significant; only *Ascaridia* sp.: *χ* ^2^ = 4.15, df = 1, *P* = 0.042, Bonferroni not significant) (Fig. 3). The odds ratio for *Egeu*‐DAB2*05 was 0.16 (all helminths) and 0.11 (only *Ascaridia* sp.), and individuals with that allele had 2.28‐ and 1.97‐fold higher chance of belonging to the category “uninfected” than individuals without that allele, respectively (*P *<* *0.05). Furthermore, individuals carrying *Egeu*‐DAB2*05 had significantly lower HMI values (*Z *= −2.45, *P *=* *0.01, Bonferroni significant) and FEC values (all helminths: *t* = 1.98, *P *=* *0.049, Bonferroni not significant; only *Ascaridia* sp.: *t *=* *2.02, *P *=* *0.044, Bonferroni not significant), compared with other individuals (Fig. 4).

### Test for the heterozygote advantage hypothesis

To test for the *heterozygote advantage hypothesis*, the effect of heterozygosity within each single locus on the individual parasite load was analyzed separately. For the three DAB loci, all comparisons indicated no effects of heterozygosity on the infection status (all helminths or only *Ascaridia* sp.: all *χ* ^2^ tests, *P *>* *0.05), the HMI values (all *χ* ^2^ tests, *P *>* *0.05), or the FEC values (all helminths or only *Ascaridia* sp.: all analyses of variance [ANOVAs], *P *>* *0.05). Furthermore, allelic divergence, an additional index of the heterozygosity, did not correlate with parasite resistance at any DAB locus regarding the infection status (all helminths or only *Ascaridia* sp.: all *t* tests, *P *>* *0.05), the HMI values (all Spearman's correlation tests, *P *>* *0.05), or the FEC values (all helminths or only *Ascaridia* sp.: all Spearman's correlation tests, *P *>* *0.05).

### Test for the allele counting hypothesis

**Figure 5:**
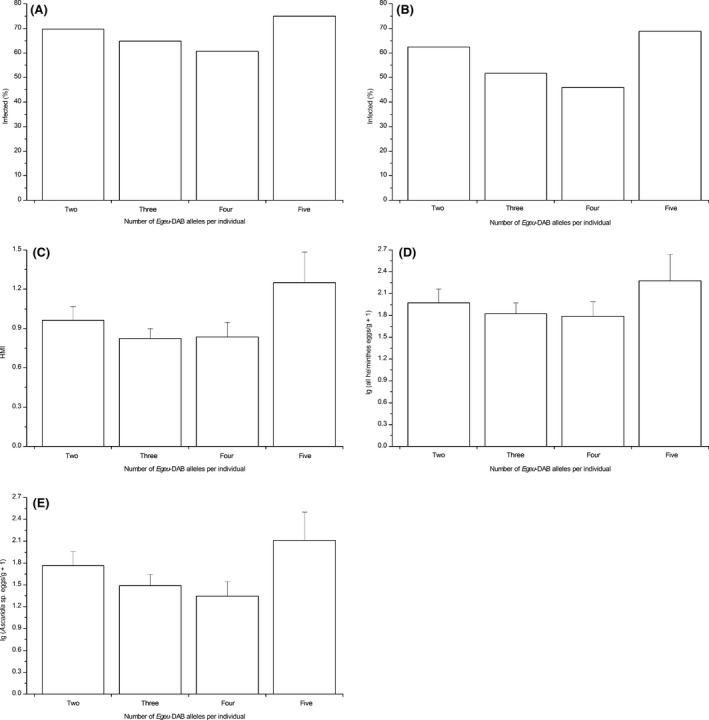
Differences in the infection measurements with respect to the number of different alleles per individual. The infection measurements are calculated as the percentage of individuals infected by (A) at least one helminth or (B) only the most abundant morphotype *Ascaridia* sp., (C) the number of helminth morphotypes per individual (HMI), the fecal egg counts (FEC, eggs/g) of (D) all helminths and (E) only *Ascaridia* sp. Means ± SE are given.

To test for the *allele counting hypothesis*, the three single DAB loci were considered as a whole (duplicated DAB loci), and then, the effect of the number of different alleles of the duplicated DAB loci per individual (two, three, four, and five, Fig. 2) on the individual parasite load was analyzed. Although the number of different alleles per individual had no significant effect on the values of percentage of individuals infected by at least one helminth or only *Ascaridia* sp. (both *χ* ^2^ tests, *P *>* *0.05), HMI (*χ* ^2^ test, *P *>* *0.05), or FEC (all helminths or only *Ascaridia* sp.: both ANOVAs, *P *>* *0.05), the values displayed consistent U‐shaped trends in all cases (Fig. 5). As shown in Figure 5, these values tended to be consistently lower in individuals with an intermediate number of alleles (three or four), compared with individuals carrying either a minimal (two) or maximal (five) number of alleles.

## Discussion

In this study, we first characterized the intestinal parasite load in the vulnerable Chinese egret. Intestinal parasites may have negative fitness consequences on wildlife populations, and most wild animals are simultaneously infected with more than one parasite species (Telfer et al. 2010; Froeschke and Sommer 2012; Kamath et al. 2014). Three diversity indices of intestinal parasites in the vulnerable Chinese egret (richness: 10, diversity: 1.14, and evenness: 0.50) were similar to those in another vulnerable bird, *Grus monacha* (8–11, 1.340–1.571, and 0.571–0.691, respectively) (Huang et al. 2014). In the population of our study, more than half of the individuals (65.63%) were found to be infected, suggesting that the Chinese egret is particularly susceptible to parasite infection. The high level of susceptibility could be explained by the potential risk factors including colonial breeding (dense population), migratory living, and relatively diverse foraging of the Chinese egret (Shiina et al. 2004; Radwan et al. 2010a; Fang et al. 2011; Petric et al. 2011; Huang et al. 2014; Zhang et al. 2015). Most of infected individuals (82.31%) in the Chinese egret were parasitized by *Ascaridia* sp., mainly because the horizontal transmission of *Ascaridia* sp. among individuals was enhanced by the close proximity of nests and frequent nest switching of fledglings in the colony (Alcaide et al. 2010). However, no sex‐dependent differences could be found in the Chinese egret either regarding the infection status, the HMI values, nor the FEC values. This finding does not support the prediction that the sex is often cited as an influencing factor for parasite infection in an animal (Poulin 1996).

The vulnerable Chinese egret had a relatively low number of MHC DAB exon 2 alleles, which is in accordance with previous reports that the levels of MHC variation, generally, are much lower in endangered species (Marsden et al. 2009; Radwan et al. 2010a,b). However, both the nucleotide and amino acid sequences of these MHC DAB exon 2 alleles showed relatively high levels of divergence at the PBRs of all the three DAB loci. The higher variability and the more nonsynonymous substitutions in the PBRs were clear indications for positive selection (Hughes and Nei 1988) and characteristics for proteins with antigen‐presenting function (Bergström and Gyllensten 1995). Concerted evolution is a molecular process that leads to a high degree of sequence similarity among multiple copies of genes within species, and in an extreme case of this evolutionary pattern, different genes share identical alleles (Wittzell et al. 1999; Miller and Lambert 2004; Li et al. 2011; Lei et al. 2015). The finding that up to 14 pairs of identically shared alleles existed in the three DAB genes further verified the concerted evolution occurred in MHC DAB genes of the Chinese egret, as proposed by our previous study (Lei et al. 2015). Sequencing multiple loci in PCR approach should be not relevant for identical alleles shared among the three DAB loci because for all loci, always one to two alleles were identified per individual (never exceed two alleles, see genotypes in Table S1), suggesting that for each locus only one gene copy was sequenced with the primer sets. Moreover, significant heterozygote deficits for all the three loci were discovered in the Chinese egret. One of the most common explanations for heterozygote deficit is the presence of null alleles (Hagell et al. 2013). However, this explanation is unlikely for the Chinese egret because all loci were specially amplified utilizing the highly conservative intron sequences flanking exon 2 (Canal et al. 2010; Hagell et al. 2013; Wang et al. 2013; Lei et al. 2015; Zhang et al. 2015). Alternatively, these deficits might be explained by inbreeding and some forms of nonrandom mating in this Chinese egret population.

In testing for the *maintenance hypotheses* of MHC polymorphism, the Chinese egret had a negative association between MHC‐specific allele and parasite load, which is similar to previous reports on other bird species, including *P. domesticus* (Loiseau et al. 2008), *G. trichas* (Dunn et al. 2013), *C. caeruleus* (Westerdahl et al. 2013), and *Z. capensis* (Jones et al. 2015). In this egret, the allele *Egeu*‐DAB2*05 was significantly linked to uninfected individuals, and individuals carrying *Egeu*‐DAB2*05 had significantly lower HMI values and FEC values. These data provided new evidence for the *rare‐allele advantage hypothesis*, which predicts that an individual with a rare MHC allele might respond better to a new parasite variant, and then this resistant allele will increase in frequency within a population (Takahata and Nei 1990). However, parasites may evolve to escape the recognition by this specific allele when it becomes common, and at last the previous resistant allele even becomes a “susceptible” allele with disadvantages for the host. The rare allele *Egeu*‐DAB2*05 (allele frequency: 0.022, Table 3) in the Chinese egret might be an example of a new emerging allele which still maintained high parasite resistance (Froeschke and Sommer 2005). In future studies, it will be necessary to clarify whether the allele frequency of *Egeu*‐DAB2*05 can be changed in this cycling pattern, as predicted by the *rare‐allele advantage hypothesis* (Froeschke and Sommer 2005), or this allele frequency is dropped because Egeu‐DAB2*05 is associated with an increased susceptibility.

Prediction of the *heterozygote advantage hypothesis* was not supported in our study. The heterozygosity within each DAB locus in the Chinese egret was not correlated with the infection status, the HMI or FEC values. To increase the level of evidence in support of the *heterozygous advantage hypothesis*, many researchers have suggested that the studies should combine two or more pathogens, as most previous studies only considered a single pathogen (e.g., virus, bacteria, or helminth) (Langefors et al. 2001; Meyer‐Lucht and Sommer 2005; Schad et al. 2005; Froeschke and Sommer 2012; Kamath et al. 2014). In the Chinese egret, although a total of 10 helminth morphotypes were investigated, none of the comparisons revealed any evidence for the *heterozygote advantage hypothesis*. The fact that no heterozygous advantage could be found in this egret may be related to the effect of the significant deviation from Hardy–Weinberg equilibrium due to the heterozygote deficit found within each locus (Zhang et al. 2015).

Our studies to test for the *allele counting hypothesis* in the Chinese egret discovered that although the number of different alleles in the duplicated DAB loci per individual had no significant effect on the values of the infection status, the HMI or the FEC, these infection values tended to be consistently lower in individuals with an intermediate number of alleles, compared with individuals carrying either a minimal or maximal number. Accordingly, these results might verify and support the *allele counting hypothesis*, which suggests intermediate, rather than maximal, allele numbers are associated with the minimal parasite load (Reusch et al. 2001). Because an intermediate allele number is optimal for an individual, one would expect individuals carrying an intermediate number of alleles to be the most frequent type within a population. As postulated, we found that egrets with an intermediate number of alleles (in this case, three and four) were the most frequent type (67.86%, Fig. 2) in the studied population. In contrast, only a few (7.14%) individuals were found to contain the maximal allele number (five), reflecting the consequence of selective disadvantages of individuals carrying larger allele numbers (Nowak et al. 1992; Harf and Sommer 2005; Milinski 2006; Woelfing et al. 2009). Our new findings provide the first evidence in support of the *allele counting hypothesis* associated MHC alleles with intestinal parasites in a bird, as within birds this hypothesis has only been reported for the associations among MHC alleles and blood parasites (e.g., Bonneaud et al. 2006; Dunn et al. 2013; Rivero‐de Aguilar et al. 2016).

In conclusion, the polymorphism of MHC *Egeu*‐DAB gene in the Chinese egret is associated with the intestinal parasite load and maintained by pathogen‐driven selection which might operate through both the *rare‐allele advantage* and the *allele counting strategy*. For this vulnerable species, the resistant allele, *Egeu*‐DAB2*05, and/or the intermediate (three or four) number of DAB alleles could be good indicators of better resistance to helminth diseases (optimal parasite resistance). In future studies, it will be necessary to further examine the role of MHC DAB gene in other ardeid birds, and investigate the parasite resistant function of other class II genes (e.g., DRA, DPB, and DQB) in the Chinese egret, because parasite antigens are typically presented by various MHC class II proteins, which may lead to a more comprehensive insight into the adaptive selection and systematic evolution of MHC in this egret and other birds.

## Data Accessibility

Genotypes of all individuals at each DAB locus are indicated in Table S1. Amino acid alignment of the 25 confirmed that MHC DAB exon 2 sequences is indicated in Figure S1. DNA sequences are available from GenBank, Accession Nos KP729234–KP729243, KP729246–KP729257, and KP729260–KP729262.

## Conflict of Interest

None declared.

## Supporting information


**Table S1.** Genotyping data collected from the three *Egeu*‐DAB loci in the Chinese egret.
**Figure S1.** Amino acid alignment of the 25 confirmed MHC DAB exon 2 sequences.

## References

[ece32226-bib-0001] Alcaide, M. , S. V. Edwards , J. J. Negro , D. Serrano , and J. L. Tella . 2008 Extensive polymorphism and geographical variation at a positively selected MHC class II B gene of the lesser kestrel (*Falco naumanni*). Mol. Ecol. 17:2652–2665. 18489548 10.1111/j.1365-294X.2008.03791.x

[ece32226-bib-0002] Alcaide, M. , J. A. Lemus , G. Blanco , J. L. Tella , D. Serrano , J. J. Negro , et al. 2010 Retracted: MHC diversity and differential exposure to pathogens in kestrels (Aves: *Falconidae*). Mol. Ecol. 19:691–705. 20074317 10.1111/j.1365-294X.2009.04507.x

[ece32226-bib-0003] Alcaide, M. , J. Muñoz , J. Martínez‐de la Puente , R. Soriguer , and J. Figuerola . 2014 Extraordinary MHC class II B diversity in a non‐passerine, wild bird: the Eurasian Coot *Fulica atra* (Aves: Rallidae). Ecol. Evol. 4:688–698. 24683452 10.1002/ece3.974 PMC3967895

[ece32226-bib-0004] Bergström, T. , and U. Gyllensten . 1995 Evolution of MHC class II polymorphism, the rise and fall of class II gene function in primates. Immunol. Rev. 143:13–31. 7558074 10.1111/j.1600-065x.1995.tb00668.x

[ece32226-bib-0005] Bernatchez, L. , and C. Landry . 2003 MHC studies in nonmodel vertebrates, what have we learned about natural selection in 15 years? J. Evol. Biol. 16:363–377. 14635837 10.1046/j.1420-9101.2003.00531.x

[ece32226-bib-0006] BirdLife International . 2015 Species factsheet: Egretta eulophotes. Available at http://www.birdlife.org.

[ece32226-bib-0007] Bodmer, W. 1972 Evolutionary significance of the HLA system. Nature 237:139–145. 4113158 10.1038/237139a0

[ece32226-bib-0008] Bonneaud, C. , J. Mazuc , O. Chastel , H. Westerdahl , and G. Sorci . 2004 Terminal investment induced by immune challenge and fitness traits associated with major histocompatibility complex in the house sparrow. Evolution 58:2823–2830. 15696759 10.1111/j.0014-3820.2004.tb01633.x

[ece32226-bib-0009] Bonneaud, C. , J. Pe′Rez‐Tris , P. Federici , O. Chastel , and G. Sorci . 2006 Major histocompatibility alleles associated with local resistance to malaria in a passerine. Evolution 60:383–389. 16610328

[ece32226-bib-0010] Brown, J. H. , T. S. Jardetzky , J. C. Gorga , L. J. Stern , R. G. Urban , J. L. Strominger , et al. 1993 Three‐dimensional structure of the human class II histocompatibility antigen HLA‐DR1. Nature 364:33–39. 8316295 10.1038/364033a0

[ece32226-bib-0011] Canal, D. , M. Alcaide , J. A. Anmarkrud , and J. Potti . 2010 Towards the simplification of MHC typing protocols, targeting classical MHC class II genes in a passerine, the pied flycatcher (*Ficedula hypoleuca*). BMC Res. Notes 3:236. 20815923 10.1186/1756-0500-3-236 PMC2944132

[ece32226-bib-0012] Clarke, B. , and D. R. Kirby . 1966 Maintenance of histocompatibility polymorphisms. Nature 211:999–1000. 6007869 10.1038/211999a0

[ece32226-bib-0013] Doherty, P. C. , and R. M. Zinkernagel . 1975 Enhanced immunological surveillance in mice heterozygous at H‐2 gene complex. Nature 256:50–52. 1079575 10.1038/256050a0

[ece32226-bib-0014] Dunn, P. O. , J. L. Bollmer , C. R. Freeman‐Gallant , and L. A. Whittingham . 2013 MHC variation is related to a sexually selected ornament, survival, and parasite resistance in common yellowthroats. Evolution 67:679–687. 23461319 10.1111/j.1558-5646.2012.01799.x

[ece32226-bib-0015] Eizaguirre, C. , T. L. Lenz , M. Kalbe , and M. Milinski . 2012 Rapid and adaptive evolution of MHC genes under parasite selection in experimental vertebrate populations. Nat. Commun. 3:621. 22233631 10.1038/ncomms1632 PMC3272583

[ece32226-bib-0016] Evans, M. L. , and B. D. Neff . 2009 Major histocompatibility complex heterozygote advantage and widespread bacterial infections in populations of Chinook salmon (*Oncorhynchus tshawytscha*). Mol. Ecol. 18:4716–4729. 19821902 10.1111/j.1365-294X.2009.04374.x

[ece32226-bib-0017] Excoffier, L. , and H. E. Lischer . 2010 Arlequin suite ver 3.5: a new series of programs to perform population genetics analyses under Linux and Windows. Mol. Ecol. Resour. 10:564–567. 21565059 10.1111/j.1755-0998.2010.02847.x

[ece32226-bib-0018] Fang, W. , Q. Lin , X. Chen , and J. Lin . 2011 Nestling diet of the vulnerable Chinese egret on offshore islands in southern China. Waterbirds 34:246–251.

[ece32226-bib-0019] Frank, S. A. 2002 Immunology and the evolution of infectious disease. Princeton Univ. Press, Princeton, NJ. 20821852

[ece32226-bib-0020] Froeschke, G. , and S. Sommer . 2005 MHC Class II DRB variability and parasite load in the striped mouse (*Rhabdomys pumilio*) in the southern Kalahari. Mol. Biol. Evol. 22:1254–1259. 15703235 10.1093/molbev/msi112

[ece32226-bib-0021] Froeschke, G. , and S. Sommer . 2012 Insights into the complex associations between MHC class II DRB polymorphism and multiple gastrointestinal parasite infestations in the striped mouse. PLoS ONE 7:e31820. 22389675 10.1371/journal.pone.0031820 PMC3289624

[ece32226-bib-0022] Godot, V. , S. Harraga , I. Beurton , P. Tiberghien , E. Sarciron , B. Gottstein , et al. 2000 Resistance/susceptibility to *Echinococcus multilocularis* infection and cytokine profile in humans. II. Influence of the HLA B8, DR3, DQ2 haplotype. Clin. Exp. Immunol. 121:491–498. 10971516 10.1046/j.1365-2249.2000.01309.x PMC1905739

[ece32226-bib-0023] Goudet, J. 1995 FSTAT, a program to estimate and test gene diversities and fixation indices (version 1.2). J. Hered. 86:485–486.

[ece32226-bib-0024] Hagell, S. , A. V. Whipple , and C. L. Chambers . 2013 Population genetic patterns among social groups of the endangered Central American spider monkey (*Ateles geoffroyi*) in a human‐dominated landscape. Ecol. Evol. 3:1388–1399. 23762523 10.1002/ece3.547 PMC3678491

[ece32226-bib-0025] Hall, T. A. 1999 BioEdit: an user‐friendly biological sequence alignment editor and analysis program for Windows 95/98/NT. Nucleic Acids Symp. Ser. 41:95–98.

[ece32226-bib-0026] Harf, R. , and S. Sommer . 2005 Association between major histocompatibility complex class II DRB alleles and parasite load in the hairy‐footed gerbil (*Gerbillurus paeba*) in the southern Kalahari. Mol. Ecol. 14:85–91. 15643953 10.1111/j.1365-294X.2004.02402.x

[ece32226-bib-0027] Hedrick, P. W. 2002 Pathogen resistance and genetic variation at MHC loci. Evolution 56:1902–1908. 12449477 10.1111/j.0014-3820.2002.tb00116.x

[ece32226-bib-0028] Hess, C. M. , and S. V. Edwards . 2002 The evolution of the major histocompatibility complex in birds. Bioscience 52:423–431.

[ece32226-bib-0029] Huang, W. , L. Zhou , and N. Zhao . 2014 Temporal‐spatial patterns of intestinal parasites of the Hooded Crane (*Grus monacha*) wintering in lakes of the middle and lower Yangtze River floodplain. Avian Resour. 5:6.

[ece32226-bib-0030] Hughes, A. L. , and M. K. Hughes . 1995 Natural selection on the peptide binding regions of major histocompatibility complex molecules. Immunogenetics 42:233–243. 7672817 10.1007/BF00176440

[ece32226-bib-0031] Hughes, A. L. , and M. Nei . 1988 Pattern of nucleotide substitution at major histocompatibility complex class I loci reveals over dominant selection. Nature 335:167–170. 3412472 10.1038/335167a0

[ece32226-bib-0032] Hughes, A. L. , and M. Yeager . 1998 Natural selection at major histocompatibility complex loci of vertebrates. Annu. Rev. Genet. 32:415–434. 9928486 10.1146/annurev.genet.32.1.415

[ece32226-bib-0033] IUCN . 2015 IUCN red list of threatened species. Available from http://www.iucnredlist.org.

[ece32226-bib-0034] Jeffery, K. J. , and C. R. Bangham . 2000 Review: do infectious diseases drive MHC diversity? Microbes Infect. 2:1335–1341. 11018450 10.1016/s1286-4579(00)01287-9

[ece32226-bib-0035] Jones, M. R. , Z. A. Cheviron , and M. D. Carling . 2015 Spatially variable coevolution between a haemosporidian parasite and the MHC of a widely distributed passerine. Ecol. Evol. 5:1045–1060. 25798222 10.1002/ece3.1391 PMC4364819

[ece32226-bib-0036] Kamath, P. L. , W. C. Turner , M. Küsters , and W. M. Getz . 2014 Parasite‐mediated selection drives an immunogenetic trade‐off in plains zebras (*Equus quagga*). Proc. R. Soc. Lond. B Biol. Sci. 281:20140077. 10.1098/rspb.2014.0077 PMC3996612 24718761

[ece32226-bib-0037] Klein, J. 1986 Natural history of the major histocompatibility complex. Wiley & Sons, New York, NY.

[ece32226-bib-0038] Klein, J. , R. E. Bontrop , R. L. Dawkins , H. A. Erlich , U. B. Gyllensten , E. R. Heise , et al. 1990 Nomenclature for major histocompatibility complexes of different species, a proposal. Immunogenetics 31:217–219. 2329006 10.1007/BF00204890

[ece32226-bib-0039] Klein, J. , Y. Satta , C. O'hUigin , and N. Takahata . 1993 The molecular descent of the major histocompatibility complex. Annu. Rev. Immunol. 11:269–295. 8476562 10.1146/annurev.iy.11.040193.001413

[ece32226-bib-0040] Kloch, A. , W. Babik , A. Bajer , E. Si?ski , and J. Radwan . 2010 Effects of an MHC‐DRB genotype and allele number on the load of gut parasites in the bank vole (*Myodes glareolus*). Mol. Ecol. 19:255–265. 20331784 10.1111/j.1365-294X.2009.04476.x

[ece32226-bib-0041] Kushlan, J. A. , and J. A. Hancock . 2005 The herons. Oxford Univ. Press, Oxford, U.K.

[ece32226-bib-0042] Langefors, A. , J. Lohm , M. Grahn , O. Andersen , and T. von Schantz . 2001 Association between major histocompatibility complex class IIB alleles and resistance to *Aeromonas salmonicida* in Atlantic salmon. Proc. R. Soc. Lond. B Biol. Sci. 268:479–485. 10.1098/rspb.2000.1378 PMC1088630 11296859

[ece32226-bib-0043] Lei, W. , W. Fang , Q. Lin , X. Zhou , and X. Chen . 2015 Characterization of a non‐classical MHC class II gene in the vulnerable Chinese egret (*Egretta eulophotes*). Immunogenetics 67:463–472. 26033691 10.1007/s00251-015-0846-1

[ece32226-bib-0044] Li, L. , X. Zhou , and X. Chen . 2011 Characterization and evolution of MHC class II B genes in ardeid birds. J. Mol. Evol. 72:474–483. 21590337 10.1007/s00239-011-9446-3

[ece32226-bib-0045] Loiseau, C. , R. Zoorob , S. Garnier , J. Birard , P. Federici , R. Julliard , et al. 2008 Antagonistic effects of a MHC class I allele on malaria‐infected house sparrows. Ecol. Lett. 11:258–265. 18070099 10.1111/j.1461-0248.2007.01141.x

[ece32226-bib-0046] Marsden, C. D. , B. K. Mable , R. Woodroffe , G. S. A. Rasmussen , S. Cleaveland , J. W. McNutt , et al. 2009 Highly endangered African wild dogs (*Lycaon pictus*) lack variation at the major histocompatibility complex. J. Hered. 100:S54–S65.

[ece32226-bib-0047] McClelland, E. E. , D. J. Penn , and W. K. Potts . 2003 Major histocompatibility complex heterozygote superiority during coinfection. Infect. Immun. 71:2079–2086. 12654829 10.1128/IAI.71.4.2079-2086.2003 PMC152037

[ece32226-bib-0048] Meyer‐Lucht, Y. , and S. Sommer . 2005 MHC diversity and the association to nematode parasitism in the yellow‐necked mouse (*Apodemus flavicollis*). Mol. Ecol. 14:2233–2243. 15910340 10.1111/j.1365-294X.2005.02557.x

[ece32226-bib-0049] Milinski, M. 2006 The major histocompatibility complex, sexual selection, and mate choice. Annu. Rev. Ecol. Evol. Syst. 37:159–186.

[ece32226-bib-0050] Miller, H. C. , and D. M. Lambert . 2004 Gene duplication and gene conversion in class II MHC genes in New Zealand robins (*Petroicidae*). Immunogenetics 56:178–191. 15138734 10.1007/s00251-004-0666-1

[ece32226-bib-0051] Nei, M. , and T. Gojobori . 1986 Simple methods for estimating the numbers of synonymous and nonsynonymous nucleotide substitutions. Mol. Biol. Evol. 3:418–426. 3444411 10.1093/oxfordjournals.molbev.a040410

[ece32226-bib-0052] Nei, M. , and A. P. Rooney . 2005 Concerted and birth‐and‐death evolution of multigene families. Annu. Rev. Genet. 39:121–152. 16285855 10.1146/annurev.genet.39.073003.112240 PMC1464479

[ece32226-bib-0053] Niskanen, A. K. , L. J. Kennedy , M. Ruokonen , I. Kojola , H. Lohi , M. Isomursu , et al. 2014 Balancing selection and heterozygote advantage in major histocompatibility complex loci of the bottlenecked Finnish wolf population. Mol. Ecol. 23:875–889. 24382313 10.1111/mec.12647

[ece32226-bib-0054] Nowak, M. A. , K. Tarczy‐Hornoch , and J. M. Austyn . 1992 The optimal number of major histocompatibility complex molecules in an individual. Proc. Natl Acad. Sci. USA 89:10896–10899. 1438295 10.1073/pnas.89.22.10896 PMC50449

[ece32226-bib-0055] O'Brien, S. J. , and J. F. Evermann . 1998 Interactive influence of infectious disease and genetic diversity in natural populations. Trends Ecol. Evol. 3:254–259. 21227241 10.1016/0169-5347(88)90058-4 PMC7134056

[ece32226-bib-0056] Ohta, T. 1991 Role of diversifying selection and gene conversion in evolution of major histocompatibility complex loci. Proc. Natl Acad. Sci. USA 88:6716–6720. 1862096 10.1073/pnas.88.15.6716 PMC52159

[ece32226-bib-0057] Oliver, M. K. , S. Telfer , and S. B. Piertney . 2009 Major histocompatibility complex (MHC) heterozygote superiority to natural multi‐parasite infections in the water vole (*Arvicola terrestris*). Proc. R. Soc. Lond. B Biol. Sci. 276:1119–1128. 10.1098/rspb.2008.1525 PMC2679068 19129114

[ece32226-bib-0058] Osborne, A. J. , J. Pearson , S. S. Negro , B. L. Chilvers , M. A. Kennedy , and N. J. Gemmell . 2015 Heterozygote advantage at MHC DRB may influence response to infectious disease epizootics. Mol. Ecol. 24:1419–1432. 25728376 10.1111/mec.13128

[ece32226-bib-0059] Paterson, S. , and M. E. Viney . 2002 Host immune responses are necessary for density dependence in nematode infections. Parasitology 125:283–292. 12358425 10.1017/s0031182002002056

[ece32226-bib-0060] Paterson, S. , K. Wilson , and J. M. Pemberton . 1998 Major histocompatibility complex variation associated with juvenile survival and parasite resistance in a large unmanaged ungulate population (*Ovis aries* L.). Proc. Natl Acad. Sci. USA 95:3714–3719. 9520432 10.1073/pnas.95.7.3714 PMC19902

[ece32226-bib-0061] Penn, D. J. 2002 The scent of genetic compatibility: sexual selection and the major histocompatibility complex. Ethology 108:1–21.

[ece32226-bib-0062] Petric, M. , I. Mladineo , and S. K. Sifner . 2011 Insight into the short‐finned Squid *Illex Coindetii* (Cephalopoda: Ommastrephidae) feeding ecology: is there a link between helminth parasites and food composition? J. Parasitol. 97:55–62. 21348607 10.1645/GE-2562.1

[ece32226-bib-0063] Piertney, S. B. , and M. K. Oliver . 2006 The evolutionary ecology of the major histocompatibility complex. Heredity 96:7–21. 16094301 10.1038/sj.hdy.6800724

[ece32226-bib-0064] Poulin, R. 1996 Sexual inequalities in helminth infections: a cost of being a male? Am. Nat. 147:287–295.

[ece32226-bib-0065] Poulin, R. 1999 The functional importance of parasites in animal communities: many roles at many levels? Int. J. Parasitol. 29:903–914. 10480727 10.1016/s0020-7519(99)00045-4

[ece32226-bib-0066] Radwan, J. , A. W. Demiaszkiewicz , R. Kowalczyk , J. Lachowicz , A. Kawalko , J. M. Wójcik , et al. 2010a An evaluation of two potential risk factors, MHC diversity and host density, for infection by an invasive nematode *Ashworthius sidemi* in endangered European bison (*Bison bonasus*). Biol. Conserv. 143:2049–2053.

[ece32226-bib-0067] Radwan, J. , A. Biedrzycka , and W. Babik . 2010b Does reduced MHC diversity decrease viability of vertebrate populations? Biol. Conserv. 143:537–544. 10.1016/j.biocon.2009.07.026 PMC7092871 32226082

[ece32226-bib-0068] Radwan, J. , M. Zagalska‐Neubauer , M. Cichoń , J. Sendecka , K. Kulma , L. Gustafsson , et al. 2012 MHC diversity, malaria and lifetime reproductive success in collared flycatchers. Mol. Ecol. 21:2469–2479. 22512812 10.1111/j.1365-294X.2012.05547.x

[ece32226-bib-0069] Reusch, T. B. H. , M. A. Häberli , P. B. Aeschlimann , and M. Milinski . 2001 Female sticklebacks count alleles in a strategy of sexual selection explaining MHC polymorphism. Nature 414:300–302. 11713527 10.1038/35104547

[ece32226-bib-0070] Rivero‐de Aguilar, J. , H. Westerdahl , J. Martínez‐de la Puente , G. Tomás , J. Martínez , and S. Merino . 2016 MHC‐I provides both quantitative resistance and susceptibility to blood parasites in blue tits in the wild. J. Avian Biol. 47:1–9.

[ece32226-bib-0071] Rousset, F. 2008 GENEPOP'007: a complete re‐implementation of the GENEPOP software for Windows and Linux. Mol. Ecol. Resour. 8:103–106. 21585727 10.1111/j.1471-8286.2007.01931.x

[ece32226-bib-0072] Sachs, I. 1992 Angewandte statistik, siebente völlig neu bearbeitete Auflage. Springer Verlag, Berlin.

[ece32226-bib-0073] Schad, J. , J. U. Ganzhorn , and S. Sommer . 2005 Parasite burden and constitution of major histocompatibility complex in the Malagasy mouse lemur (*Microcebus murinus*). Evolution 59:439–450. 15807428

[ece32226-bib-0074] Schad, J. , D. K. N. Dechmann , C. C. Voigt , and S. Sommer . 2012 Evidence for the ‘good genes’ model: association of MHC class II DRB alleles with ectoparasitism and reproductive state in the Neotropical Lesser Bulldog Bat, *Noctilio albiventris* . PLoS ONE 7:e37101. 22615910 10.1371/journal.pone.0037101 PMC3353892

[ece32226-bib-0075] Schwensow, N. , J. Fietz , K. Dausmann , and S. Sommer . 2007 Neutral versus adaptive genetic variation in parasite resistance: importance of MHC‐supertypes in a free‐ranging primate. Heredity 99:265–277. 17519969 10.1038/sj.hdy.6800993

[ece32226-bib-0076] Shiina, T. , S. Shimizu , K. Hosomichi , S. Kohara , S. Watanabe , K. Hanzawa , et al. 2004 Comparative genomic analysis of two avian (quail and chicken) MHC regions. J. Immunol. 172:6751–6763. 15153492 10.4049/jimmunol.172.11.6751

[ece32226-bib-0077] Sin, Y. W. , G. Annavi , H. L. Dugdale , C. Newman , T. Burke , and D. W. MacDonald . 2014 Pathogen burden, co‐infection and major histocompatibility complex variability in the European badger (*Meles meles*). Mol. Ecol. 23:5072–5088. 25211523 10.1111/mec.12917

[ece32226-bib-0078] Sloss, M. W. , R. L. Kemp , and A. Zajac . 1994 Veterinary clinical parasitology. 6th ed State Univ. Press, Ames, IA.

[ece32226-bib-0079] Sommer, S. 2005 The importance of immune gene variability (MHC) in evolutionary ecology and conservation. Front. Zool. 2:16. 16242022 10.1186/1742-9994-2-16 PMC1282567

[ece32226-bib-0080] Stear, M. J. , K. Bairden , J. L. Duncan , P. H. Holmes , Q. A. McKellar , M. Park , et al. 1997 How hosts control worms. Nature 389:27. 9288962 10.1038/37895

[ece32226-bib-0081] Takahata, N. , and M. Nei . 1990 Allelic genealogy under overdominant and frequency‐dependent selection and polymorphism of major histocompatibility complex loci. Genetics 124:967–978. 2323559 10.1093/genetics/124.4.967 PMC1203987

[ece32226-bib-0082] Tamura, K. , G. Stecher , D. Peterson , A. Filipski , and S. Kumar . 2013 MEGA6: Molecular Evolutionary Genetics Analysis version 6.0. Mol. Biol. Evol. 30:2725–2729. 24132122 10.1093/molbev/mst197 PMC3840312

[ece32226-bib-0083] Telfer, S. , X. Lambin , R. Birtles , P. Beldomenico , S. Burthe , S. Paterson , et al. 2010 Species interactions in a parasite community drive infection risk in a wildlife population. Science 330:243–246. 20929776 10.1126/science.1190333 PMC3033556

[ece32226-bib-0084] Valilou, R. H. , S. A. Rafat , D. R. Notter , D. Shojda , G. Moghaddam , and A. Nematollahi . 2015 Fecal egg counts for gastrointestinal nematodes are associated with a polymorphism in the MHC‐DRB1 gene in the Iranian Ghezel sheep breed. Front. Genet. 6:105. 25852746 10.3389/fgene.2015.00105 PMC4371757

[ece32226-bib-0085] Wang, Z. , X. Zhou , Q. Lin , W. Fang , and X. Chen . 2011 New primers for sex identification in the Chinese egret and other ardeid species. Mol. Ecol. Resour. 11:176–179. 21429119 10.1111/j.1755-0998.2010.02879.x

[ece32226-bib-0086] Wang, Z. , X. Zhou , Q. Lin , W. Fang , and X. Chen . 2013 Characterization, polymorphism and selection of major histocompatibility complex (MHC) DAB genes in vulnerable Chinese egret (*Egretta eulophotes*). PLoS ONE 8:e74185. 24019955 10.1371/journal.pone.0074185 PMC3760844

[ece32226-bib-0087] Wegner, K. M. , M. Kalbe , J. Kurtz , T. B. H. Reusch , and M. Milinski . 2003a Parasite selection for immunogenetic optimality. Science 301:1343. 12958352 10.1126/science.1088293

[ece32226-bib-0088] Wegner, K. M. , T. B. H. Reusch , and M. Kalbe . 2003b Multiple parasites are driving major histocompatibility complex polymorphism in the wild. J. Evol. Biol. 16:224–232. 14635861 10.1046/j.1420-9101.2003.00519.x

[ece32226-bib-0089] Westerdahl, H. , M. Asghar , D. Hasselquist , and S. Bensch . 2012 Quantitative disease resistance: to better understand parasite‐mediated selection on major histocompatibility complex. Proc. R. Soc. Lond. B Biol. Sci. 279:577–584. 10.1098/rspb.2011.0917 PMC3234550 21733902

[ece32226-bib-0090] Westerdahl, H. , M. Stjernman , L. Raberg , M. Lannefors , and J.‐A. Nilsson . 2013 MHC‐I affects infection intensity but not infection status with a frequent avian malaria parasite in Blue tits. PLoS ONE 8:e72647. 24023631 10.1371/journal.pone.0072647 PMC3758318

[ece32226-bib-0091] Wittzell, H. , A. Bernot , C. Auffrey , and R. Zoorob . 1999 Concerted evolution of two Mhc class II B locus in pheasants and domestic chickens. Mol. Biol. Evol. 16:479–490. 10331274 10.1093/oxfordjournals.molbev.a026130

[ece32226-bib-0092] Woelfing, B. , A. Traulsen , M. Milinski , and T. Boehm . 2009 Does intra‐individual major histocompatibility complex diversity keep a golden mean? Philos. Trans. R. Soc. Lond. B Biol. Sci. 364:117–128. 18926972 10.1098/rstb.2008.0174 PMC2666699

[ece32226-bib-0093] Worley, K. , J. Collet , E. G. Spurgin , C. Cornwallis , T. Pizzari , and D. S. Richardson . 2010 MHC heterozygosity and survival in red junglefowl. Mol. Ecol. 19:3064–3075. 20618904 10.1111/j.1365-294X.2010.04724.x

[ece32226-bib-0094] Zhang, M. , and H. He . 2013 Parasite‐mediated selection of major histocompatibility complex variability in wild Brandt's voles (*Lasiopodomys brandtii*) from Inner Mongolia, China. BMC Evol. Biol. 13:149. 23848494 10.1186/1471-2148-13-149 PMC3720540

[ece32226-bib-0095] Zhang, L. , Q. Wu , Y. Hu , H. Wu , and F. Wei . 2015 Major histocompatibility complex alleles associated with parasite susceptibility in wild giant pandas. Heredity 114:85–93. 25248466 10.1038/hdy.2014.73 PMC4815596

[ece32226-bib-0096] Zhou, X. , W. Fang , and X. Chen . 2010 Mitochondrial DNA diversity of the vulnerable Chinese Egret (*Egretta eulophotes*) from China. J. Ornithol. 151:409–414.

