# Supplementary material for: Major histocompatibility complex class II DAB alleles associated with intestinal parasite load in the vulnerable Chinese egret (Egretta eulophotes)
Source: Ecol Evol. 2016 Jun 7;6(13):4421–34. doi: 10.1002/ece3.2226 (PMC4930990; doi:10.1002/ece3.2226)
Supplement: Supplementary file 1 — Table S1. Genotyping data collected from the three Egeu‐DAB loci in the Chinese egret. Figure S1. Amino acid alignment of the 25 confirmed MHC DAB exon 2 sequences. [file ECE3-6-4421-s001.doc]

**Table S1** Genotyping data collected from the three *Egeu*-DAB loci in the Chinese egret

| Individual | *Egeu*-DAB1 | *Egeu*-DAB2 | *Egeu*-DAB3 |
| --- | --- | --- | --- |
| Ee001 | 0303^a^ | 0101 | 0101 |
| Ee002 | 0606 | 0303 | 0101 |
| Ee003 | 0606 | 0102 | 0102 |
| Ee004 | 0203 | 0102 | 0101 |
| Ee005 | 0203 | 0102 | 0101 |
| Ee006 | 0101 | 0104 | 0101 |
| Ee007 | 0303 | 0107 | 0101 |
| Ee008 | 0203 | 0102 | 0101 |
| Ee009 | 0505 | 0102 | 0101 |
| Ee010 | 0505 | 0102 | 0101 |
| Ee011 | 0106 | 0105 | 0303 |
| Ee012 | 0101 | 0102 | 0103 |
| Ee013 | 0203 | 0102 | 0102 |
| Ee014 | 0303 | 0101 | 0102 |
| Ee015 | 0106 | 0309 | 0202 |
| Ee016 | 0303 | 0101 | 0102 |
| Ee017 | 0303 | 0101 | 0102 |
| Ee018 | 0303 | 0101 | 0101 |
| Ee019 | 0203 | 0102 | 0101 |
| Ee020 | 0303 | 0101 | 0102 |
| Ee021 | 0101 | 0102 | 0101 |
| Ee022 | 0105 | 0102 | 0303 |
| Ee023 | 0303 | 0101 | 0101 |
| Ee024 | 0101 | 0404 | 0101 |
| Ee025 | 0101 | 0104 | 0103 |
| Ee026 | 0303 | 0101 | 0101 |
| Ee027 | 0303 | 0101 | 0101 |
| Ee028 | 0606 | 0101 | 0101 |
| Ee029 | 0606 | 0101 | 0101 |
| Ee030 | 0506 | 0202 | 0101 |
| Ee031 | 0506 | 0202 | 0102 |
| Ee032 | 0606 | 0101 | 0101 |
| Ee033 | 0101 | 0104 | 0103 |
| Ee034 | 0203 | 0102 | 0101 |
| Ee035 | 0606 | 0303 | 0101 |
| Ee036 | 0203 | 0102 | 0101 |
| Ee037 | 0106 | 0309 | 0101 |
| Ee038 | 0910 | 1112 | 0102 |
| Ee039 | 0110 | 0212 | 0101 |
| Ee040 | 0505 | 0102 | 0101 |
| Ee041 | 0606 | 0303 | 0202 |
| Ee042 | 0101 | 0404 | 0202 |
| Ee043 | 0101 | 0405 | 0303 |
| Ee044 | 0203 | 0102 | 0101 |
| Ee045 | 0101 | 0105 | 0103 |
| Ee046 | 0303 | 0101 | 0101 |
| Ee047 | 0303 | 0101 | 0101 |
| Ee048 | 0106 | 0101 | 0101 |
| Ee049 | 0303 | 0101 | 0101 |
| Ee050 | 0303 | 0101 | 0101 |
| Ee051 | 0203 | 0102 | 0101 |
| Ee052 | 0203 | 0102 | 0101 |
| Ee053 | 0606 | 0101 | 0101 |
| Ee054 | 0203 | 0102 | 0101 |
| Ee055 | 0101 | 0104 | 0101 |
| Ee056 | 0101 | 0104 | 0101 |
| Ee057 | 0203 | 0102 | 0101 |
| Ee058 | 0303 | 0101 | 0101 |
| Ee059 | 0303 | 0101 | 0101 |
| Ee060 | 0303 | 0101 | 0101 |
| Ee061 | 0303 | 0101 | 0101 |
| Ee062 | 0303 | 0101 | 0102 |
| Ee063 | 0203 | 0104 | 0101 |
| Ee064 | 0203 | 0102 | 0202 |
| Ee065 | 0101 | 0104 | 0101 |
| Ee066 | 0101 | 0104 | 0101 |
| Ee067 | 0303 | 1010 | 0102 |
| Ee068 | 0606 | 0101 | 0101 |
| Ee069 | 0101 | 0405 | 0303 |
| Ee070 | 0106 | 0404 | 0103 |
| Ee071 | 0101 | 0104 | 0101 |
| Ee072 | 0101 | 0104 | 0101 |
| Ee073 | 0303 | 0101 | 0101 |
| Ee074 | 0505 | 0102 | 0101 |
| Ee075 | 0505 | 0102 | 0101 |
| Ee076 | 0505 | 0202 | 0202 |
| Ee077 | 0303 | 0101 | 0101 |
| Ee078 | 0303 | 0101 | 0101 |
| Ee079 | 0101 | 0102 | 0101 |
| Ee080 | 0203 | 0102 | 0103 |
| Ee081 | 0101 | 0102 | 0103 |
| Ee082 | 0101 | 0404 | 0102 |
| Ee083 | 0106 | 0404 | 0102 |
| Ee084 | 0106 | 0404 | 0102 |
| Ee085 | 0106 | 0404 | 0103 |
| Ee086 | 0106 | 0102 | 0303 |
| Ee087 | 0606 | 0207 | 0103 |
| Ee088 | 0101 | 0404 | 0101 |
| Ee089 | 0101 | 0104 | 0101 |
| Ee090 | 0101 | 0404 | 0202 |
| Ee091 | 0101 | 0404 | 0202 |
| Ee092 | 0101 | 0104 | 0101 |
| Ee093 | 0203 | 0102 | 0101 |
| Ee094 | 0203 | 0102 | 0101 |
| Ee095 | 0303 | 0101 | 0101 |
| Ee096 | 0303 | 0101 | 0101 |
| Ee097 | 0303 | 0101 | 0101 |
| Ee098 | 0505 | 0102 | 0101 |
| Ee099 | 0203 | 0102 | 0101 |
| Ee100 | 0203 | 0102 | 0101 |
| Ee101 | 0506 | 0202 | 0101 |
| Ee102 | 0505 | 0102 | 0101 |
| Ee103 | 0505 | 0102 | 0101 |
| Ee104 | 0303 | 0101 | 0101 |
| Ee105 | 0101 | 0505 | 0203 |
| Ee106 | 0303 | 0101 | 0102 |
| Ee107 | 0203 | 0102 | 0101 |
| Ee108 | 0203 | 0102 | 0101 |
| Ee109 | 0606 | 0101 | 0101 |
| Ee110 | 0101 | 0102 | 0101 |
| Ee111 | 0606 | 0101 | 0101 |
| Ee112 | 0606 | 0101 | 0101 |
| Ee113 | 0303 | 0101 | 0101 |
| Ee114 | 0303 | 0101 | 0101 |
| Ee115 | 0101 | 0102 | 0202 |
| Ee116 | 0303 | 0101 | 0101 |
| Ee117 | 0303 | 0101 | 0101 |
| Ee118 | 0303 | 0101 | 0101 |
| Ee119 | 0505 | 0102 | 0101 |
| Ee120 | 0404 | 0107 | 0101 |
| Ee121 | 0106 | 0101 | 0101 |
| Ee122 | 0106 | 0309 | 0101 |
| Ee123 | 0203 | 0102 | 0101 |
| Ee124 | 0505 | 0102 | 0101 |
| Ee125 | 0303 | 0101 | 0101 |
| Ee126 | 0910 | 0211 | 0102 |
| Ee127 | 0101 | 0104 | 0101 |
| Ee128 | 0606 | 0101 | 0101 |
| Ee129 | 0606 | 0101 | 0101 |
| Ee130 | 0106 | 0505 | 0303 |
| Ee131 | 0106 | 0102 | 0101 |
| Ee132 | 0101 | 0101 | 0101 |
| Ee133 | 0101 | 0101 | 0101 |
| Ee134 | 0606 | 0104 | 0101 |
| Ee135 | 0106 | 0404 | 0101 |
| Ee136 | 0106 | 0404 | 0101 |
| Ee137 | 0606 | 0104 | 0101 |
| Ee138 | 0303 | 0101 | 0101 |
| Ee139 | 0303 | 0101 | 0101 |
| Ee140 | 0303 | 0101 | 0101 |
| Ee141 | 0708 | 0608 | 0303 |
| Ee142 | 0303 | 0101 | 0101 |
| Ee143 | 0303 | 0101 | 0102 |
| Ee144 | 0203 | 0102 | 0101 |
| Ee145 | 0303 | 0101 | 0101 |
| Ee146 | 0303 | 0101 | 0102 |
| Ee147 | 0101 | 0707 | 0202 |
| Ee148 | 0101 | 0707 | 0202 |
| Ee149 | 0606 | 0404 | 0102 |
| Ee150 | 0606 | 0404 | 0101 |
| Ee151 | 0303 | 0101 | 0101 |
| Ee152 | 0303 | 0101 | 0101 |
| Ee153 | 0303 | 0101 | 0101 |
| Ee154 | 0303 | 0101 | 0101 |
| Ee155 | 0303 | 0101 | 0101 |
| Ee156 | 0303 | 0101 | 0101 |
| Ee157 | 0606 | 0105 | 0103 |
| Ee158 | 0606 | 0102 | 0101 |
| Ee159 | 0303 | 0101 | 0101 |
| Ee160 | 0606 | 0102 | 0101 |
| Ee161 | 0101 | 0101 | 0101 |
| Ee162 | 0101 | 0101 | 0101 |
| Ee163 | 0606 | 0404 | 0202 |
| Ee164 | 0303 | 0101 | 0101 |
| Ee165 | 0303 | 0101 | 0101 |
| Ee166 | 0606 | 0104 | 0101 |
| Ee167 | 0303 | 0101 | 0101 |
| Ee168 | 0606 | 0404 | 0101 |
| Ee169 | 0303 | 0101 | 0101 |
| Ee170 | 0303 | 0101 | 0101 |
| Ee171 | 0303 | 0101 | 0101 |
| Ee172 | 0606 | 0404 | 0101 |
| Ee173 | 0203 | 0104 | 0101 |
| Ee174 | 0203 | 0102 | 0101 |
| Ee175 | 0303 | 0101 | 0101 |
| Ee176 | 0303 | 0101 | 0101 |
| Ee177 | 0203 | 0102 | 0101 |
| Ee178 | 0101 | 0101 | 0101 |
| Ee179 | 0101 | 0101 | 0101 |
| Ee180 | 0101 | 0101 | 0101 |
| Ee181 | 0203 | 0102 | 0101 |
| Ee182 | 0303 | 0101 | 0101 |
| Ee183 | 0106 | 0309 | 0101 |
| Ee184 | 0606 | 0404 | 0101 |
| Ee185 | 0303 | 0101 | 0101 |
| Ee186 | 0303 | 0101 | 0101 |
| Ee187 | 0203 | 0102 | 0101 |
| Ee188 | 0106 | 0309 | 0101 |
| Ee189 | 0606 | 0404 | 0101 |
| Ee190 | 0303 | 0101 | 0101 |
| Ee191 | 0304 | 0101 | 0101 |
| Ee192 | 0506 | 0202 | 0102 |
| Ee193 | 0303 | 0101 | 0101 |
| Ee194 | 0203 | 0102 | 0101 |
| Ee195 | 0506 | 0202 | 0303 |
| Ee196 | 0606 | 0104 | 0101 |
| Ee197 | 0505 | 0202 | 0202 |
| Ee198 | 0505 | 0102 | 0101 |
| Ee199 | 0106 | 0101 | 0101 |
| Ee200 | 0505 | 0102 | 0101 |
| Ee201 | 0303 | 0101 | 0101 |
| Ee202 | 0303 | 0101 | 0101 |
| Ee203 | 0303 | 0101 | 0101 |
| Ee204 | 0203 | 0102 | 0101 |
| Ee205 | 0203 | 0102 | 0101 |
| Ee206 | 0606 | 0104 | 0101 |
| Ee207 | 0606 | 0105 | 0103 |
| Ee208 | 0203 | 0102 | 0101 |
| Ee209 | 0303 | 0101 | 0102 |
| Ee210 | 0303 | 0101 | 0101 |
| Ee211 | 0203 | 0102 | 0101 |
| Ee212 | 0101 | 0102 | 0303 |
| Ee213 | 0101 | 0102 | 0101 |
| Ee214 | 0303 | 0101 | 0102 |
| Ee215 | 0303 | 0101 | 0102 |
| Ee216 | 0203 | 0102 | 0101 |
| Ee217 | 0106 | 0101 | 0101 |
| Ee218 | 0106 | 0102 | 0101 |
| Ee219 | 0106 | 0101 | 0101 |
| Ee220 | 0203 | 0102 | 0101 |
| Ee221 | 0505 | 0102 | 0101 |
| Ee222 | 0106 | 0101 | 0101 |
| Ee223 | 0106 | 0101 | 0101 |
| Ee224 | 0505 | 0102 | 0101 |

^a^ Two sequential allele numbers (01–12).


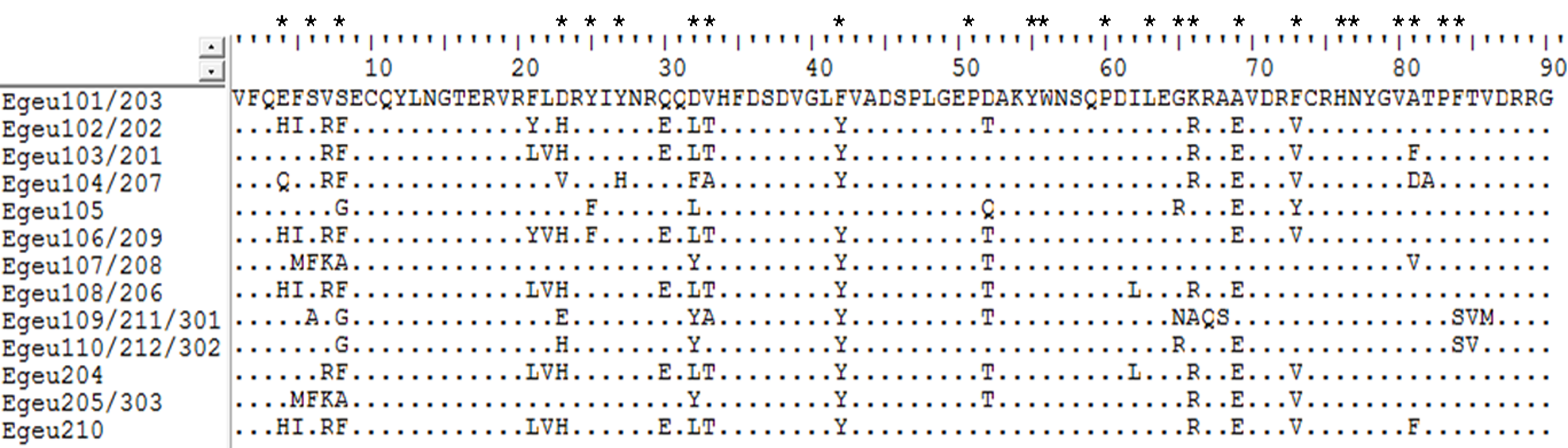


**Fig. S1** Amino acid alignment of the 25 confirmed MHC DAB exon 2 sequences. The alleles are denoted by the species’ gene prefix (*Egeu*) with a suffix comprising a locus number (1–3) and two sequential allele numbers (01–12). The fourteen pairs of identically shared alleles among the three DAB loci are indicated in the *leftmost* column by the same rows. *Dots* represent identity with the top sequence; *asterisks* indicate putative peptide-binding regions, based on analyses of Brown *et al*. (1993).
